# Antimicrobial and Anti-Infective Activity of Natural Products—Gaining Knowledge from Novel Studies

**DOI:** 10.3390/antibiotics12061051

**Published:** 2023-06-15

**Authors:** Elizabeth S. Fernandes, Isabella F. da Silva Figueiredo, Cinara R. A. V. Monteiro, Valério Monteiro-Neto

**Affiliations:** 1 Programa de Pós-Graduação em Biotecnologia Aplicada à Saúde da Criança e do Adolescente, Faculdades Pequeno Príncipe, Av. Iguaçu No 333, Curitiba 80230-020, PR, Brazil; bellaafigueiredo@hotmail.com; 2 Instituto de Pesquisa Pelé Pequeno Príncipe, Av. Silva Jardim No 1632, Curitiba 80240-020, PR, Brazil; 3 Programa de Pós-Graduação em Ciências da Saúde, Universidade Federal do Maranhão, Av. dos Portugueses No 1966, Cidade Universitária Dom Delgado, São Luís 65085-040, MA, Brazil; cinaraaragao@hotmail.com (C.R.A.V.M.); valerio.monteiro@ufma.br (V.M.-N.)

## Abstract

Despite advances in the development of antimicrobial drugs in the last centuries, antimicrobial resistance has consistently raised in the last decades, compromising their effectiveness. Novel antimicrobial compounds, especially from natural sources, including plants, microorganisms, and animals, have since become a growing area of research. In this context, studies covering the investigation of their ability to combat resistant microorganisms, either by neutralization or inactivation of pathogen resistance mechanisms and virulence properties, have gained attention. Herein, a collection of 19 manuscripts focused on the antimicrobial and anti-infective activity of natural products, including their mechanisms of action, in silico evidence of antimicrobial activity, synergistic associations with antibiotics, and other aspects, will be discussed.

Of the published papers, 15 are original research studies, three are reviews (one is a systematic review of the literature), and one is a brief report. Kopel and collaborators [1] reviewed and discussed the in vitro models and clinical trials for assessing the antimicrobial activities of phytochemicals. They highlighted the great diversity of compounds, such as alkaloids, organosulfur compounds, phenols, coumarins, and terpenes found in plants, as well as their broad spectrum of antimicrobial activity and resistance. Amongst the novel compounds discussed are the alkaloids sanguinarine, found in *Sanguinaria canadensis,* and tomatidine, found in solanaceous plants, such as tomatoes and potatoes. These were shown to interfere with bacterial cell division and cytokinesis through disruption of the plasma membrane, increased cell permeabilization, and inhibition of protein synthesis; enhancement of reactive oxygen species generation and reduction in ergosterol formation by fungi were also discussed as mechanisms of action of the alkaloids. They also commented on the ability of these compounds, as well as of other alkaloids, to potentiate the action of commercial antimicrobials, such as vancomycin, streptomycin, and ciprofloxacin. Organosulfur compounds, such as allicin from raw garlic, isothiocyanates, including sulforaphane, and allyl, benzyl, and phenethyl isothiocyanates were all suggested to act against a range of microorganisms, presenting antimicrobial and anti-virulence activities, which may be equivalent or even stronger than common antibiotics. Their mechanisms of action varied from disruption of cell walls, interference with essential biochemical pathways, and inhibition of enzymes, amongst others. Phenolic compounds, such as flavonoids (e.g., galangin, kaempferol quercetin, catechins, etc.) act on bacterial enzymes and toxins, disturbing cytoplasmic membranes, preventing the formation of biofilms, alone or in synergy with wide spectrum antibiotics. All the above-described mechanisms of action were first demonstrated by means of in vitro assays, such as microdilution and agar well diffusion methods, biofilm and hyphae formation techniques, and biochemical studies. Promising results on the in vitro antimicrobial actions of natural compounds can evolve to clinical trials to assess the effects of plant extract-based mouthwashes, gels, creams, suppositories, as well as other formulations for systemic usage, for a variety of diseases. Finally, the authors highlighted the need for further randomized clinical trials with large populations of subjects to evaluate the efficacy of natural products, in addition to the importance of regulations and quality standards for developing efficient and affordable phytochemicals as alternative to antibiotics.

Another review by Araújo et al. [2] introduced surface-active compounds—biomolecules produced by microorganisms (e.g., *Acinetobacter* sp., *Bacillus* sp., *Candida* sp., *Lactobacillus* sp., *Pseudomonas* sp., *Serratia* sp.)—able to interact with surfaces and hydrophobic or hydrophilic interfaces. Biosurfactants and emulsifiers are included in this group of compounds, and their actions as antimicrobials, modulators of virulence factors, anticancer, and wound healing agents were discussed. Biosurfactants can be classified as glycolipids, lipopeptides, lipoproteins or fatty acids, and phospholipid polymers. Their chemical compositions vary according with the producer microorganism—which can be found in different types of water and land, and even in extreme conditions of contaminants, temperatures, pH values, and salinity levels. The most abundant are glycolipids and lipopeptides. Bioemulsifiers comprehend composites of heteropolysaccharides, lipopolysaccharides, proteins, glycoproteins, or lipoproteins; and they can be isolated from contaminated soil, mangroves, seawater, freshwater, and human skin. These surface-active compounds have applications in different industrial sectors, such as pharmaceutical, textile, agriculture, cosmetics, personal care, and food industries, as well as for environmental purposes in soil remediation, hydrocarbon degradation, and oil recovery. Their antimicrobial and antibiofilm actions are due to the ability of breaking the outer and inner membranes of pathogens, reducing replication, inhibition of cell adhesion, and blocking of the invasion of host cells by harmful microorganisms. Surface-active compounds are also able to counteract inflammation by reducing neutrophil migration and inflammatory mediator release, as well as by modulating lymphocyte populations. Additional anti-cancer properties include apoptosis and impairment of the replication of cancer cells, whilst pro-healing effects may occur via numerous pathways that regulate the synthesis of collagenases, metalloproteinases, growth factors, and cytokines; this results in promoting fibroblast and epithelial cell proliferation, re-epithelialization, collagen deposition, and thus, faster healing. The authors emphasized the suitability of biosurfactants and bioemulsifiers as candidates for future biotechnological, biomedical, and pharmaceutical applications.

A systematic review by De La Hoz-Romo and collaborators [3] explored the potential of marine actinobacteria-derived compounds for the treatment of acne vulgaris, a multifactorial disease, which has been associated with microbial dysbiosis and inflammation of the pilosebaceous unit. *Cutibacterium acnes* is the most abundant microorganism of the pilosebaceous unit, followed by *Staphylococcus epidermidis*; however, these may become opportunistic pathogens involved in skin dysbiosis. In this context, antimicrobials are important tools for disease management. Marine actinobacteria represent an interesting source of diverse compounds, with antibacterial, antibiofilm, anticoagulant, antiviral, and antibacterial effects. In their manuscript, the authors performed a systematic analysis of metabolites and extracts produced by marine actinobacteria with antimicrobial, anti-biofilm, and quorum-sensing inhibition activities (quorum quenching, QQ), as therapeutic alternatives treatment of acne vulgaris, some skin diseases, and infectious diseases. They also classified them in clusters and associated these with their corresponding biosynthetic genes, considering their structure–activity relationship. They found that most of the studies on the anti-infective activity of marine actinobacteria-derived compounds were from China, India, and Egypt. Their sources were marine sediments, sponges, and other marine invertebrates, such as sea squirts, corals, echinoderm-derived organisms, mollusks, and jellyfish, as well as marine algae, water, mangroves, seagrasses, and fishes. The genuses most reported were *Streptomyces*, *Nocardiopsis*, *Micromonospora*, *Salinospora*, and *Verruscosispora*. These bacterial crude extracts and compounds were effective against *Staphylococcus* sp., inhibiting its growth and biofilm formation. Amongst the compounds identified in the bacterial crude extracts (most of them from *Streptomyces* sp.) were flavonoids, citreamicins, anthracyclines, chromomycins, napyradiomycins, marinomycins, and kokumarin. Then, the strategies to maximize the anti-infective activity and yield of metabolites were discussed by the authors; they found that starch and yeast extract peptone are the best sources of carbon and nitrogen, respectively, to maximize the production of anti-infective compound from cultured marine actinobacteria. Finally, the compounds were stratified into compounds with antibacterial activity and compounds with QQ activity; different biosynthetic gene clusters were identified and associated with the compounds. The results emphasize the antimicrobial potential of these marine bacterial compounds and the need for novel studies in the field.

Four of the original research studies investigated novel antifungals derived from plants. Mendonça et al. [4] demonstrated, by using in silico and in vitro analysis, that the ethyl acetate fraction obtained from the leaves of *P. granatum* and its isolated compound, galloyl-hexahydroxydiphenoyl-glucose (a hydrolysable tannin), are effective against *Candida albicans* and *C. glabrata*, inhibiting their growth and biofilm formation. Both the fraction and the compound were able to potentiate the effects of fluconazole—a fungistatic usually used to treat yeast infections. *P. granatum* fraction, as well as galloyl-hexahydroxydiphenoyl-glucose, reduced phospholipase production by *Candida* sp., an important virulence factor of fungi. Finally, the study highlights the importance of *P. granatum* as a source of antimicrobials and the need for further studies with the tannin identified in the study. In another report by Motta et al. [5], *C. albicans* and *C. glabrata* were incubated with the hydroalcoholic extract from the leaves of *Vismia guianensis*, a native Brazilian plant. The extract showed antifungal and fungicidal activities against *Candida* sp., inhibiting fungi growth and adhesion, as well as disrupting both early and mature biofilms. Fourteen compounds were identified in the extract, with a predominance of anthraquinones, flavonoids, and vismione D. In silico analysis predicted a high probability for vismione D to act as an antifungal and an anti-inflammatory. On the other hand, kaempferol and quercetin exhibited the highest predictive value as anti-inflammatories, and quercetin and catechin had the highest values as antioxidant compounds. By molecular docking, the authors determined possible interactions between *V. guianensis* compounds and *C. albicans* CaCYP51—a cytochrome P450 enzyme required for the biosynthesis of sterols. Vismione D presented the highest predicted affinity in relation to CaCYP51. The interesting results indicate the need for further investigations with *V. guianensis* and its compounds, especially in regards of *Candida*-induced diseases. Silva and colleagues [6] assessed the antifungal potential of sulforaphane, an isothiocyanate found in cruciferous plants, with previous suggested antibacterial actions. The authors conducted both in silico analysis and in vitro assays; as expected, in silico analysis confirmed its antimicrobial properties, which include antiparasitic, antifungal, and antibacterial actions, as well as mechanisms ranging from alterations of membrane composition to inhibition of fungi RNA. The compound was estimated to present moderate risk for mutagenic, tumorigenic, and reproductive tract deleterious effects. The analysis also indicated that sulforaphane has a greater potential to permeate the skin than fluconazole, suggesting its potential use to treat superficial mycosis. In vitro assays with *Candida* sp. ATCC strains and clinical isolates from the oral cavity and vaginal smears demonstrated the ability of the compound to inhibit fungi growth, as well as hyphae and biofilm formation. Additional tests revealed that sulforaphane can potentiate fluconazole antifungal actions when assessed at sub-inhibitory concentrations. Similar results on hyphae and biofilm formation by *C. albicans* were seen when the compounds were combined. These effects were related to their ability to interfere with the expression of hyphae growth-related and biofilm formation-related genes by *C. albicans*, although this may vary according with strains and isolates. The authors conclude that the association of sulforaphane and fluconazole may be beneficial to treat *Candida* spp. infections and may trigger fewer adverse reactions caused by these compounds. They also discuss the importance of additional research to address the effects of their combination on mixed populations of microorganisms, including those found in the oral cavity and the vagina. In another study, Leesombun et al. [7] investigated the antifungal activity of the essential oil and ethanolic extract of *Coleus amboinicus* against *Microsporum canis*—a zoophilic dermatophyte found in domestic cats and dogs, which can also cause disease in humans. The activity of the essential oil and extract of *C. amboinicus* was assessed in vitro against clinical isolates obtained from feline samples, classified as weak, moderate, and strong biofilm-producers. In order to evaluate their effects on biofilm formation, minimum inhibitory concentrations (MICs) were determined and, then, the microorganisms were incubated with MIC and 2 × MIC for 96 h. Both the essential oil and the extract inhibited biofilm formation by *M. canis*. Similar inhibitory effects on growth and biofilm formation were observed when these preparations were assessed against *C. albicans* and *Trichophyton rubrum*. Chemical analysis revealed the presence of 18 compounds in the essential oil, including carvacrol (major compound), p-cymene, and γ-terpinene, which are all monoterpenes. The flavonoids caffeic acid, rosmarinic acid, and apigenin were detected in *C. amboinicus* extract. These results highlight the antifungal potential of *C. amboinicus* formulations for treating zoonotic infections caused by *M. canis*.

The antimicrobial and anti-parasitic actions of extracts and compounds obtained from fungi and bacteria were analysed in three of the of the original research studies. The first, performed by Mazzone and collaborators [8], explored the in vitro activity of natural products from *Paraboeremia selaginellae*—an endophytic fungus against *Toxoplasma gondii*. They isolated eight natural products from the crude extract of *P. selaginellae* found in the leaves of *Philodendron monstera*—an ornamental plant native to the Americas; six of them inhibited *T. gondii* proliferation with low or no effects on other microrganisms. Possible toxic actions of these compounds were assessed in human Hs27 fibroblast, THP-1, Huh-7, and Hek 293 cell lines, with three of them (biphenyl ether derivatives) showing no toxicity when tested at ≤100 µM. The results indicate the promising potential of endophytic fungi as sources for novel anti-toxoplasma compounds. In another investigation, Diyaolu et al. [9] isolated and identified a strain of *Aspergillus fumigatus* (UIAU-3F) from soil samples collected from the River Oyun in Kwara State in Nigeria. *A. fumigatus* was cultured in different conditions, resulting in three fungus extracts prepared and subjected to comparative metabolomics. The extract obtained from the fungus cultured in rice medium produced the greatest diversity of metabolites. Following its fractioning into two compounds, fumitremorgin C and pseurotin D were isolated. Molecular docking was performed for the compounds against two enzymes, cruzain and l-lactate dehydrogenase, from *Trypanosoma cruzi* and *Plasmodium falciparum*, respectively. The compounds were found to interact with both enzymes; their docking was comparable to the docking observed for benznidazole (an anti-trypanosoma) and chloroquine (an anti-plasmodium). When tested in vitro, fumitremorgin C presented lower efficacy than benznidazole and chloroquine, but it displayed significantly higher activities against *T. cruzi* and *P. falciparum* in comparison to pseurotin D. The lack of effects of pseurotin D was suggested to be due to its low cell permeability, interstitial hypertension, and/or metabolic degradation. This was the first study of the anti-parasitic effects of fumitremorgin C and pseurotin D, and it highlights the need for further research, especially with fumitremorgin C, to determine mechanisms of action and potential use in vivo. In the third study, Mondal and Thomas [10] isolated and characterized novel Actinomycetes found in marine sediments, and they assessed its antimicrobial activity against fish pathogens. Sixteen (16) different Actinomycete colonies were obtained from marine sediment samples from the coast of Digha in India. The isolates were then tested against two fish pathogens—*Aeromonas hydrophila* and *Vibrio parahemolyticus*. Two of them presented antibacterial activity, with *Beijerinckia fluminensis* VIT01 being the most potent. Of note, this study reported, for the first time, the isolation of *B. fluminensis* from marine sediments. This bacterium was found to be able to hydrolyze starch, gelatin, and casein, grow in salt concentrations as high as 7%, and survive in temperatures from 28–40 °C. Fourier-transform infrared spectroscopy of the crude bacterial extract revealed 14 vibrational bands, corresponding to eight groups of compounds, including thiol, carboxylic acid, isothiocyanate, tertiary alcohol, amine, alkene, 1,2-disubstituted, and fluoro and halo compounds. Gas chromatography-mass spectrometry (GC/MS) detected 18 different major compounds; of those, N, N-Dimethylheptanamide, Glycine,N-Octyl-, Ethyl Ester, Glycyl-L-Proline, Actinomycin C2, (S)-3,4-Dimethylpentanol, and 7-Tetradecene were suggested to be antibacterial metabolites. The data indicate these compounds could be used as alternative treatments for infections in aquaculture.

Eight of the original research studies investigated the antibacterial effects of plant extracts, fractions, and compounds. The study by Figueiredo et al. [11] looked at the protective effects of cinnamaldehyde—a compound known for its antimicrobial and immunomodulatory actions, in mice systemically injected with a pathogenic *Escherichia coli* (strain F5, which was isolated from the blood stream). The compound, given by gavage, at 60 mg/kg 2 h after infection, reduced mortality by 40% over five days of observation. Cinnamaldehyde was effective against *E. coli* in vitro, but not in vivo; however, septic mice treated with the compound exhibited less tissue haemorrhage, inflammation, and damage in different organs, especially in the lungs in comparison with vehicle-treated mice. Cinnamaldehyde partially reverted leukocyte counts in the peritoneal and bone marrow of septic mice. A stimulatory effect was also observed in *E. coli*-infected mice treated with the compound; however, the same animals presented with reduced circulating and peritoneal levels of inflammatory mediators (cytokines and chemokines). The study reinforces previous data on the ability of cinnamaldehyde to act as a modulator of the immune/inflammatory response in sepsis. Moraes-Neto et al. [12] investigated the antimycobacterial and anti-inflammatory activities of the ethyl acetate fraction of *Bixa orellana* leaves and one of its active compound—ellagic acid. In silico analysis indicated anti-inflammatory, antioxidant, antibacterial, antimycobacterial, and hepatoprotective activities for ellagic acid. Additionally, the compound was predicted to be better absorbed by the intestine than clarithromycin, and to not be mutagenic, tumorigenic, irritant, or harmful to the reproductive system. Molecular docking revealed that ellagic acid can bind to COX_2_ and to mycobacterial dihydrofolate reductase, indicating its potential mechanisms of anti-inflammatory and antimicrobial activities. Both ellagic acid and *B. orellana* ethyl acetate fraction were effective against *Mycobacterium abscessus* in vitro and protected infected *Tenebrio molitor* larvae, reducing their mortality when tested at MIC, 2 × MIC, and 4 × MIC. The anti-inflammatory effects of *B. orellana* fraction and ellagic acid were confirmed in a mouse model of paw inflammation induced by carragenan, in which they both reduced oedema formation. The results support the anti-inflammatory and antimicrobial potential of *B. orellana* and highlight the need for further investigations on the mechanisms of action of the plant fraction and its active compound and for broader studies with a higher number of mycobacteria strains.

Protocatechuic acid (PCA) is a plant-derived phenolic acid with known antimicrobial activity and ability to enhance antibiotic action; although a promising molecule, its low bioavailability and fast metabolism/excretion has limited its clinical usage [13]. Miklasińska-Majdanik et al. [14] used an esterified PCA—protocatechuic acid ethyl ester to gain knowledge on its antimicrobial actions against staphylococcal strains, alone or in combination with erythromycin. Twelve strains were tested in microdilution and checkerboard assays; protocatechuic acid ethyl ester reduced erythromycin MIC values by ≥50% when incubated with nine of them. Although these are preliminary results, they show a clear and potent anti-staphylococcal effect for protocatechuic acid ethyl ester in vitro. In another study, da Silva and colleagues [15] assessed the actions of solasodine—a natural steroidal alkaloid found in plants of the *Solanum* genus—against *S. aureus*, *P. aeruginosa,* and *E. coli*. They also analysed the in vitro combination effects of solasodine with gentamicin, ciprofloxacin, norfloxacin, or ampicillin, as well as the inhibitory actions of the compound on NorA and MepA multidrug efflux pumps. From the eight bacterial strains tested, solasodine had no effects on four of them. The compound was able to potentiate both gentamicin and norfloxacin activities when tested against multidrug resistant strains of *S. aureus*, *P. aeruginosa,* and *E. coli*. Ampicillin, on the other hand, was only potentiated by solasodine when incubated with *S. aureus*. The synergistic effects between solasodine and ciprofloxacin were found to be related to inhibition of the MePA efflux pump. An antagonistic effect between the compound and ampicillin or norfloxacin was also observed for some of the bacterial strains of *S. aureus* tested. The authors suggest the different effects noted may depend on the mechanism of action of the antibiotics, or even antibiotic chelation by solasodine. Nonetheless, future studies on the combination effects of plant alkaloids with antibiotics represent an interesting tool for treating bacterial infections whilst reducing resistance.

Another original report by Gazwi and collaborators showed the antimicrobial (antifungal and antibacterial), antioxidant, and cytotoxic properties of the hydroalcoholic extract from the red flowers of *Malvaviscus arboreus* Cav.—a tropical and subtropical perennial deciduous shrub native to Central and South America [16]. Phytochemical analysis of the extract by GC-MS detected 21 compounds; 11-octadecenoic acid methyl ester, 9,12-octadecadienoic acid (Z, Z)-2-hydroxy-1-(hydroxymethyl) ethyl ester, 9,12-Octadecadienoic acid (Z, Z)-methyl ester, exadecenoic acid methyl ester, hexadecanoic acid, and oleic acid were the predominant compounds. Additionally, 13 phenolic compounds were identified by high-performance liquid chromatography: naringin, hesperidin, kaempferol, luteolin, apigenin, catechin, caffeic acid, cinnamic acid, gallic acid, syringic acid, benzoic acid, and ellagic acid. Proton nuclear magnetic resonance revealed the presence of oxygenated saturated and unsaturated hydrocarbon compounds, such as saturated and unsaturated fatty acids in the extract. Different compounds detected were previously reported as antimicrobials and antioxidants. In accordance, *M. arboreus* acted as an efficient free radical scavenger and presented a strong antibacterial activity against *Vibrio damsela* and moderate activity against *V. fluvialis* and *S. typhimurium*. An antifungal property was also noted against *Aspergillus terreus*, *A. fumigatus*, and *A. flavus*. The extract exhibited a long-lasting action on *V. damsel* and was able to potentiate amoxicillin/clavulanic acid antibacterial effects on this microorganism. *M. arboreus* triggered apoptosis (via caspase 3/7 activation), autophagy, and cell cycle arrest in hepatocellular carcinoma HepG2 cells. Overall, the data indicate a promising usage of compounds from the hydroalcoholic extract from the red flowers of *M. arboreus* as anticancer, antioxidant, and antimicrobial therapies. In the study by Almutairi [17], the antimicrobial effects of frankincense (*Boswellia sacra*) oil, its interaction with imipenem and gentamicin against methicillin-resistant *S. aureus,* and multidrug-resistant *P. aeruginosa* were determined in vitro and in vivo. GC/MS analysis revealed the presence of 40 constituents. Poor antibacterial effects and no interactions with the tested antibiotics were observed when the oil was incubated with *S. aureus* or *P. aeruginosa*. No interactions between the antibiotics and the frankincense oil were observed in vivo, although the oil alone greatly attenuated bacterial load and tissue damage in the lungs of rats with *S. aureus*-induced pneumonia. The lack of potential benefits from the combination between frankincense oil and the tested antibiotics is discouraging; however, the in vivo results allow us to suggest that the oil, per se, may act as a modulator of inflammatory responses in infectious diseases. Another interesting study by Pérez-Delgado [18] explored the in vitro antibacterial activity of *Apis mellifera,* venom collected from bees of Lambayeque, northern Peru, against *E. coli*, *Pseudomonas aeruginosa*, and *Staphylococcus aureus* reference strains. Three peptides of low molecular weights (5 kDa, 6 kDa, and 7 kDa) were separated from the crude bee venom. A strong and moderate antimicrobial effect was noted against *E. coli* and *S. aureus*, respectively, whilst no effects were observed for the peptide fractions when incubated with *P. aeruginosa*. At the MIC found for *E. coli,* the fraction did not promote haemolysis, although this was noted for higher concentrations. Additionally, no antioxidant actions were detected for *A. mellifera* fraction; this was attributed to the lack of melittin—a polypeptide with anti-inflammatory and antioxidant actions [19]—in its composition. The study indicates the importance of other peptides as antimicrobials.

The beneficial role of another plant-derived preparation was investigated against *Bipolaris sorokiniana*—a fungi known to infect wheat crops. Its effects were also tested against bacteria resistant to antibiotics (*E. coli*, *Klebsiella pneumonia* and *S. aureus*). The study by Alqahtani et al. [20] demonstrated the effects of silver nanoparticles, containing *Mangifera indica* leaf extract. An important antibacterial effect was noted for the nanoparticle in vitro. When applied to diseased wheat, the nanoparticles markedly increased reduced sugar levels and total phenol content. The same plants also exhibited higher activation of the enzymes superoxide dismutase, catalase, peroxidase, and glutathione reductase, indicating protection against oxidative stress damage. The nanoparticles also enhanced the activity of phenylalanine ammonia lyase, an enzyme involved in the synthesis of secondary metabolites. Reduction in disease severity was only observed for the highest concentrations of nanoparticles (≥30 ppm). Overall, the application of silver nanoparticles on wheat crops causes may prevent *B. sorokiniana* infections.

Finally, the brief report by Heuser and colleagues [21] looked at the bactericidal activity of sodium bituminosulfonate (a substance derived from sulfur-rich oil shale) against methicillin-susceptible and methicillin-resistant clinical isolates of *S. aureus*. All 40 isolates tested were susceptible to sodium bituminosulfonate, which demonstrated a rapid bactericidal action in vitro. The results suggest this compound is a promising alternative to the available topical antibiotics.

Taken together, the manuscripts discussed herein unveiled the potential of different natural preparations and compounds from plants and microorganisms to treating infections of importance to humans, animals, and wheat. Some of them also predicted side-effects and cellular targets for the natural compounds. All these studies presented interesting highlights regarding the need for further investigations in order to uncover more in-depth information on their additional properties, safety, pharmacokinetic, and pharmacodynamic profiles.

## References

[1] Kopel J., McDonald J., Hamood A. (2022). An Assessment of the In Vitro Models and Clinical Trials Related to the Antimicrobial Activities of Phytochemicals. Antibiotics.

[2] Araujo J., Monteiro J., Silva D., Alencar A., Silva K., Coelho L., Pacheco W., Silva D., Silva M., Silva L. (2022). Surface-Active Compounds Produced by Microorganisms: Promising Molecules for the Development of Antimicrobial, Anti-Inflammatory, and Healing Agents. Antibiotics.

[3] De La Hoz-Romo M.C., Diaz L., Villamil L. (2022). Marine Actinobacteria a New Source of Antibacterial Metabolites to Treat Acne Vulgaris Disease-A Systematic Literature Review. Antibiotics.

[4] Mendonca A.M.S., Monteiro C.A., Moraes-Neto R.N., Monteiro A.S., Mondego-Oliveira R., Nascimento C.E.C., da Silva L.C.N., Lima-Neto L.G., Carvalho R.C., de Sousa E.M. (2022). Ethyl Acetate Fraction of Punica granatum and Its Galloyl-HHDP-Glucose Compound, Alone or in Combination with Fluconazole, Have Antifungal and Antivirulence Properties against *Candida* spp.. Antibiotics.

[5] Motta E.P., Farias J.R., Costa A., Silva A.F.D., Oliveira Lopes A.J., Cartagenes M., Nicolete R., Abreu A.G., Fernandes E.S., Nascimento F.R.F. (2022). The Anti-Virulence Effect of Vismia guianensis against Candida albicans and Candida glabrata. Antibiotics.

[6] Silva B.L.R., Simao G., Campos C.D.L., Monteiro C., Bueno L.R., Ortis G.B., Mendes S.J.F., Moreira I.V., Maria-Ferreira D., Sousa E.M. (2022). In Silico and In Vitro Analysis of Sulforaphane Anti-Candida Activity. Antibiotics.

[7] Leesombun A., Thanapakdeechaikul K., Suwannawiang J., Mukto P., Sungpradit S., Bangphoomi N., Changbunjong T., Thongjuy O., Weluwanarak T., Boonmasawai S. (2022). Effects of Coleus amboinicus L. Essential Oil and Ethanolic Extracts on Planktonic Cells and Biofilm Formation of Microsporum canis Isolated from Feline Dermatophytosis. Antibiotics.

[8] Mazzone F., Simons V.E., van Geelen L., Frank M., Mandi A., Kurtan T., Pfeffer K., Kalscheuer R. (2022). In Vitro Biological Activity of Natural Products from the Endophytic Fungus Paraboeremia selaginellae against Toxoplasma gondii. Antibiotics.

[9] Diyaolu O.A., Preet G., Fagbemi A.A., Annang F., Perez-Moreno G., Bosch-Navarrete C., Adebisi O.O., Oluwabusola E.T., Milne B.F., Jaspars M. (2023). Antiparasitic Activities of Compounds Isolated from Aspergillus fumigatus Strain Discovered in Northcentral Nigeria. Antibiotics.

[10] Mondal H., Thomas J. (2022). Isolation and Characterization of a Novel Actinomycete Isolated from Marine Sediments and Its Antibacterial Activity against Fish Pathogens. Antibiotics.

[11] Figueiredo I.F.S., Araujo L.G., Assuncao R.G., Dutra I.L., Nascimento J.R., Rego F.S., Rolim C.S., Alves L.S.R., Frazao M.A., Cadete S.F. (2022). Cinnamaldehyde Increases the Survival of Mice Submitted to Sepsis Induced by Extraintestinal Pathogenic Escherichia coli. Antibiotics.

[12] Moraes-Neto R.N., Coutinho G.G., Ataide A.C.S., de Oliveira Rezende A., Nascimento C.E.C., de Albuquerque R.P., da Rocha C.Q., Rego A.S., de Sousa Cartagenes M.D.S., Abreu-Silva A.L. (2022). Ethyl Acetate Fraction of Bixa orellana and Its Component Ellagic Acid Exert Antibacterial and Anti-Inflammatory Properties against Mycobacterium abscessus subsp. massiliense. Antibiotics.

[13] Kakkar S., Bais S. (2014). A review on protocatechuic Acid and its pharmacological potential. ISRN Pharmacol..

[14] Miklasinska-Majdanik M., Kepa M., Kulczak M., Ochwat M., Wasik T.J. (2022). The Array of Antibacterial Action of Protocatechuic Acid Ethyl Ester and Erythromycin on Staphylococcal Strains. Antibiotics.

[15] da Silva A.R.P., Costa M.D.S., Araujo N.J.S., de Freitas T.S., de Almeida R.S., Barbosa Filho J.M., Tavares J.F., de Souza E.O., de Farias P.A.M., Pinheiro J.C.A. (2022). Potentiation of Antibiotic Action and Efflux Pump Inhibitory Effect on *Staphylococcus aureus* Strains by Solasodine. Antibiotics.

[16] Gazwi H.S.S., Shoeib N.A., Mahmoud M.E., Soltan O.I.A., Hamed M.M., Ragab A.E. (2022). Phytochemical Profile of the Ethanol Extract of Malvaviscus arboreus Red Flower and Investigation of the Antioxidant, Antimicrobial, and Cytotoxic Activities. Antibiotics.

[17] Almutairi M.B.F., Alrouji M., Almuhanna Y., Asad M., Joseph B. (2022). In-Vitro and In-Vivo Antibacterial Effects of Frankincense Oil and Its Interaction with Some Antibiotics against Multidrug-Resistant Pathogens. Antibiotics.

[18] Perez-Delgado O., Espinoza-Culupu A.O., Lopez-Lopez E. (2023). Antimicrobial Activity of Apis mellifera Bee Venom Collected in Northern Peru. Antibiotics.

[19] Khalil A., Elesawy B.H., Ali T.M., Ahmed O.M. (2021). Bee Venom: From Venom to Drug. Molecules.

[20] Alqahtani Y.S., Bahafi A., Mirajkar K.K., Basavaraju R.R., Mitra S., Shailaja S., More S.S., Muddapur U.M., Khan A.A., Sudarshan P.R. (2022). In Vitro Antibacterial Activity of Green Synthesized Silver Nanoparticles Using Mangifera indica Aqueous Leaf Extract against Multidrug-Resistant Pathogens. Antibiotics.

[21] Heuser E., Becker K., Idelevich E.A. (2022). Bactericidal Activity of Sodium Bituminosulfonate against *Staphylococcus aureus*. Antibiotics.

